# Prediction of low-risk breast cancer using quantitative DCE-MRI and its pathological basis

**DOI:** 10.18632/oncotarget.22267

**Published:** 2017-11-01

**Authors:** Tingting Xu, Lin Zhang, Hong Xu, Sifeng Kang, Yali Xu, Xiaoyu Luo, Ting Hua, Guangyu Tang

**Affiliations:** ^1^ Department of Radiology, Tenth People’s Hospital, Tongji University School of Medicine, Shanghai, 200072, China

**Keywords:** dynamic contrast enhanced magnetic resonance imaging, low-risk breast cancer, apparent diffusion coefficient, quantitative parameters, pathological basis

## Abstract

**Purpose:**

This study aimed to evaluate the difference of mass in dynamic contrast enhanced magnetic resonance imaging (DCE-MRI) characteristics between low-risk and non-low-risk breast cancers and to explore the possible pathological basis.

**Materials and Methods:**

Approval from the institutional review board and informed consent were acquired for this study. The MR images of 104 patients with pathologically proven breast cancer (104 lesions) were prospectively analyzed. All of included patients were Chinese woman. The DCE-MRI morphologic findings, apparent diffusion coefficient (ADC) values, quantitative DCE-MRI parameters, and pathological biomarkers between the two subtypes of breast cancer were compared. The quantitative DCE-MRI parameters and ADC values were added to the morphologic features in multivariate models to evaluate diagnostic performance in predicting low-risk breast cancer. The values were further subjected to the receiver operating characteristic (ROC) curve analysis.

**Results:**

Low-risk tumors showed significantly lower *K*^trans^ and *K*_e*p*_ *value*s (*t* = 2.065, *P* = 0.043 and *t* = 3.548, *P* = 0.001, respectively) and higher ADC value (*t* = 4.713, *P* = 0.000) than non-low-risk breast cancers. Our results revealed no significant differences in clinic data and conventional imaging findings between the two breast cancer subtypes. Adding the quantitative DCE-MRI parameters and ADC values to conventional MRI improved the diagnostic performance of MRI: The area under the ROC improved from 0.63 to 0.91. Low-risk breast cancers showed significantly lower matrix metalloproteinase (MMP)-2 expression (*P* = 0.000), lower MMP-9 expression (*P* = 0.001), and lower microvessel density (MVD) values (*P* = 0.008) compared with non-low-risk breast cancers. *K*^trans^ and *K*_e*p*_ values were positively correlated with pathological biomarkers. The ADC value showed a significant inverse correlation with pathological biomarkers.

**Conclusions:**

The prediction parameter using *K*^trans^, *K*_ep_, and ADC obtained on DCE-MRI and diffusion-weighted imaging could facilitate the identification of low-risk breast cancers. Decreased biological factors, including MVD, vascular endothelial growth factor, MMP-2, and MMP-9, may explain the possible pathological basis.

## INTRODUCTION

Breast carcinoma is a heterogeneous disease that has various prognoses and treatment responses [1]. Thus, different and appropriate management is very important for subtypes of breast carcinoma. Recently, four factors, namely the estrogen receptor (ER), Human epidermal growth factor receptor 2 (HER2), as well as Ki67 are used to confirm the subtypes. These four-factors can predict long time results of hormone receptor (HR) positive breast cancers as well as guide the neoadjuvant chemotherapy [2, 3]. The tumors can be classified to non-low risk and low risk cancers according to the gene expression profiles [4, 5]. Shin HJ et al. defined ER+ tumors with low histologic grade, Ki67 lower than 15%, negative lymph node metastasis as well as HER2-negetive as low-risk breast cancer [4]. Low risk breast cancers are considered to be at low risk for recurrence, have good prognosis. Non-low-risk breast cancers are associated with aggressive histological features, poor prognosis, a high risk for recurrence, and an increased risk of death, which will benefit from adjuvant RT [6]. A personalized approach to make a precise subtype diagnosis in breast cancer before operation would be significantly valuable for the pretreatment planning and prognosis of patients.

Dynamic contrast enhanced magnetic resonance imaging (DCE-MRI) with high sensitivity and moderate specificity is considered to be the most accurate imaging technique for the diagnosis of breast tumor [7]. Diffusion-weighted imaging (DWI) can detect tissue water changes which are associated with tissue and intracellular structure, and reflects cellular density by the apparent diffusion coefficient (ADC) [8]. There are few reports on the imaging biomarkers of diagnosing indolent tumors [4, 6]. Particularly, the exact pathological basis of the difference of imaging findings between two subtypes of breast cancer was not fully researched. Therefore, this study evaluated the differences between low-risk and non-low-risk breast cancers in imaging, and explored the possible pathological basis.

## RESULTS

### Patients

The 104 patients with breast cancer were all invasive ductal carcinoma (*n* = 104). Low-risk tumors constituted 38% (40/104) cases and the other tumors constituted 62% (64/104) cases based on the classified standard proposed by Shin HJ et al. The characteristics of clinical and pathologic are shown in Table 1. The patient’s age and mass location did not differ significantly between the two subtypes. Statistically significant differences can be founded in the distribution of ER (*P* = 0.000), PR (*P* = 0.000), HER2 (*P* = 0.000) and Ki67 (*P* = 0.000) between two groups.

**Table 1:** Clinical and pathologic characteristics of breast cancer

Characteristic	Low-risk tumor (*n* = 40)	Non-low-risk tumor (*n* = 64)	*X*^*2*^	*P* value
Age^#^	57.56 (22–84)	57.40 (30–75)		0.95
tumor Location			0.470	0.976
Central area	8	12		
UOQ	14	26		
LOQ	8	13		
UIQ	7	9		
LIQ	3	4		
ER				
positive	40 (100)	24 (38)	40.625	0.000^*^
negative	0 (0)	40 (62)		
PR			29.962	0.000^*^
positive	8 (20)	48 (75)		
negative	32 (80)	16 (25)		
HER2			44.032	0.000^*^
positive	0 (0)	42 (66)		
negative	40 (100)	22 (34)		
Ki67			88.836	0.000^*^
positive	0 (0)	60 (94)		
negative	40 (100)	4 (6)		

Note – Numbers in parentheses are percentages.

Significant differences on the 0.05 level are marked by ^*^.

^#^indicating mean age and age range in parentheses.

### DCE-MRI findings of two subtypes of breast cancer

Table 2 shows that the morphologic features, such as size, shape, margin, and location, of the tumors were not statistically significant between the two subtypes. On delayed enhancement images, the difference in the enhancement characteristic of the two subtypes was either not statistically significant (*P* = 0.089). In the TIC pattern, low-risk breast cancers tended to present type I (17% vs 4%) and non-low-risk breast cancers tended to present type III (56% vs 33%). The difference between the two subtypes breast cancer was either not statistically significant (*P* = 0.054).

**Table 2:** Conventional DCE-MRI features of two subtypes of breast cancer

MRI imaging finding	Low-risk tumor (*n* = 40)	Non-low-risk tumor (*n* = 64)	χ2	*P*
Size of mass(cm)			2.654	0.265
≤ 2	10 (25)	20 (31)		
2–5	25 (63)	30 (47)		
≥ 5	5 (12)	14 (22)		
Shape of tumor			1.266	0.531
Round	5 (12)	9 (14)		
oval	12 (30)	13 (20)		
Irregular	23 (56)	42 (58)		
Margin of tumor			1.257	0.553
Smooth	8 (20)	12 (19)		
Irregular	13 (32)	15 (23)		
Spiculation	19 (48)	37 (58)		
Enhancement pattern			4.845	0.089
Homogeneous	17 (17)	15 (20)		
Heterogeneous	22 (40)	44 (28)		
Rim	1 (43)	5 (52)		
TIC type			5.830	0.054
I	10 (17)	8 (4)		
II	18 (35)	20 (30)		
III	12 (33)	36 (56)		

Note–TIC means time–signal intensity curve; n means sample size.

**Figure 1:**
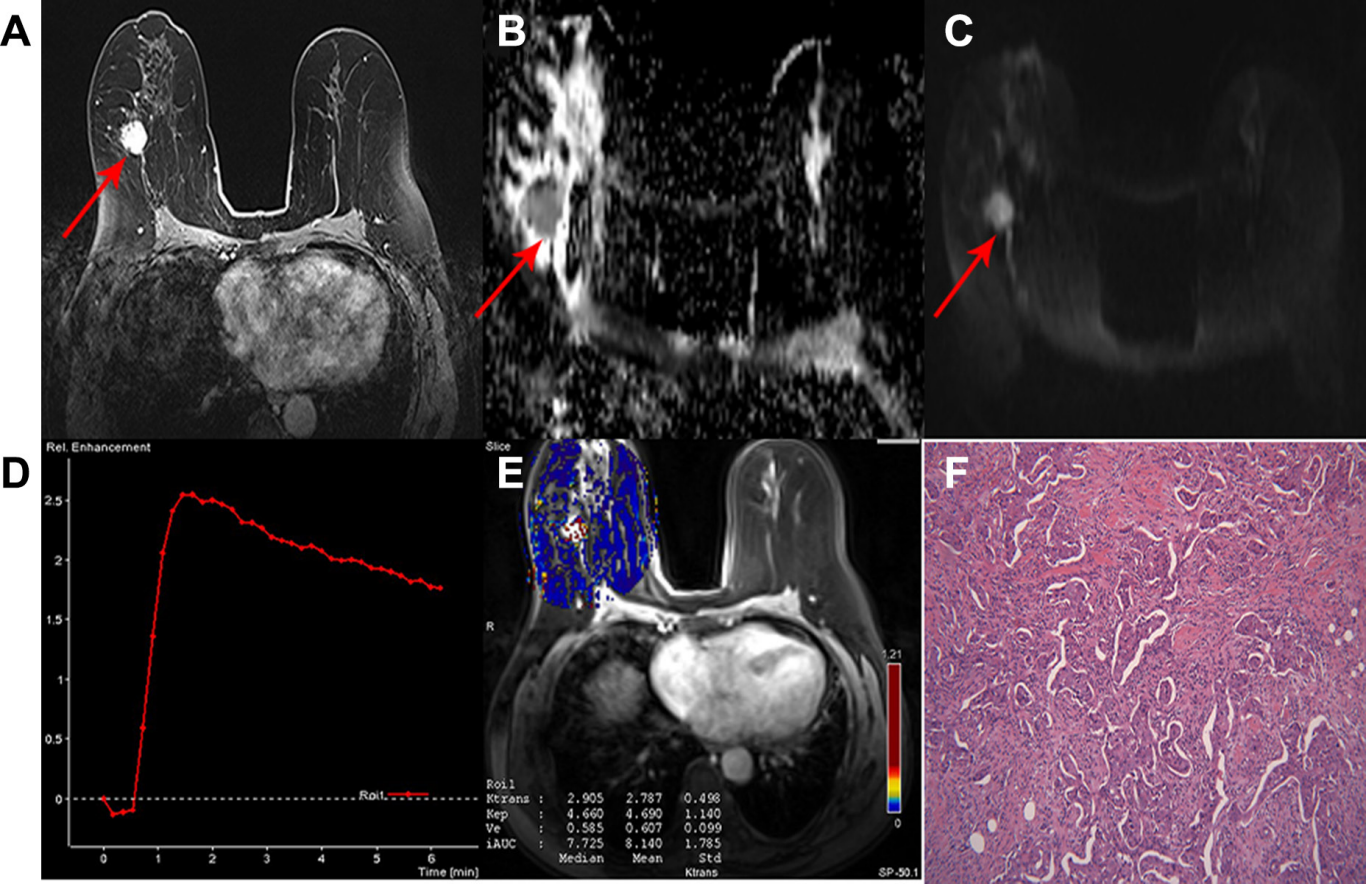
A 63-year-old woman with invasive ductal carcinoma in the right breast classified as a non-low-risk tumor (**A**) Delayed contrast enhancement image shows a tumor with rough margin, oval shape and homogeneous enhancement in the right breast. (**B**) ADC map and (**C**) DW image showing hyperintensity signal with mean ADC 0.90 × 10^−3^ mm^2^/sec. (**D**) TIC showed a tumor with type 3 curve. (**E**) Quantitative DCE-MRI images with calculated perfusion parameters (*K*^trans^ 2.787, *K*_ep_ 4.690 and Ve 0.607). (**F**) Microscopic image demonstrating the diagnosis of IDC (H&E staining, original magnification ×100).

**Figure 2:**
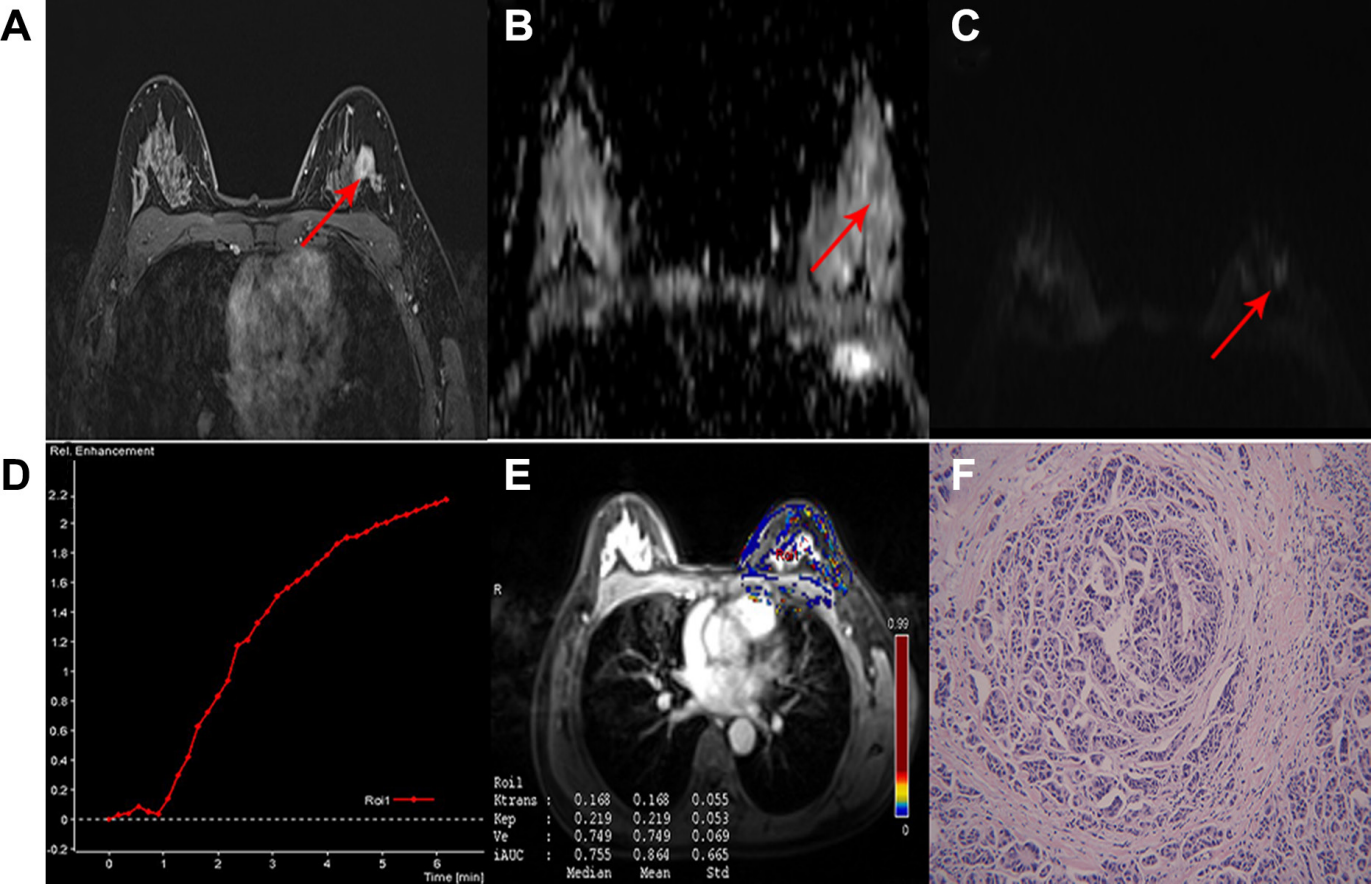
A 47-year-old woman with IDC in the left breast classified as low-risk breast cancer (**A**) Delayed contrast enhanced image, shows a tumor with smooth margin, irregular shape and heterogeneous enhancement. (**B**) ADC map and (**C**) DW image showing isointensity signal with mean ADC 1.2 × 10−3 mm2/sec on ADC map. (**D**) TIC displaying a tumor with type 1 curve. (**E**) Quantitative DCE-MRI image with calculated perfusion parameters (Ktrans 0.168, Kep 0.219, Ve 0.749). (**F**) Microscopic image demonstrating the diagnosis of IDC (H&E staining, original magnification ×100).

On univariate analysis, low-risk cancers shows obviously lower *K*^trans^ and *K*_e*p*_ values (*t* = 2.065, *P* = 0.043 and *t* = 3.548, *P* = 0.001, respectively) and higher ADC value (*t* = 4.713, *P* = 0.000) than non-low-risk breast cancers (Table 3). No obvious differences in Ve value can be observed between the two subtypes of breast cancers. Figure 1 and Figure 2 show the representative characteristics of the two subtypes.

**Table 3:** Comparison of quantitative DCE-MRI parameters and ADC values between two subtypes of breast cancer

Parameters	Low-risk tumor (*n* = 40) Mean ± SD	Non-low-risk tumor (*n* =64) Mean ± SD	*t*	*P*
*K*^trans^(min^–1^)	0.928 ± 0.630	1.275 ± 0.665	2.065	0.043^*^
*K*_ep_(min^–1^)	1.698 ± 0.980	2.919 ± 1.403	3.548	0.001^*^
Ve	0.527 ± 0.177	0.546 ± 0.152	0.448	0.656
ADC(10^−3^ mm^2^/sec)	1.056 ± 0.227	0.806 ± 0.128	4.713	0.000^*^

Note–ADC means apparent diffusion coefficient; Ktrans means volume transfer constant; Kep means rate constant between extravascular extracellular space and blood plasma; Ve means volume of extravascular extracellular space per unit volume of tissue.

SD means standard deviation.

Significant differences on the 0.05 level are marked by ^*^.

**Figure 3:**
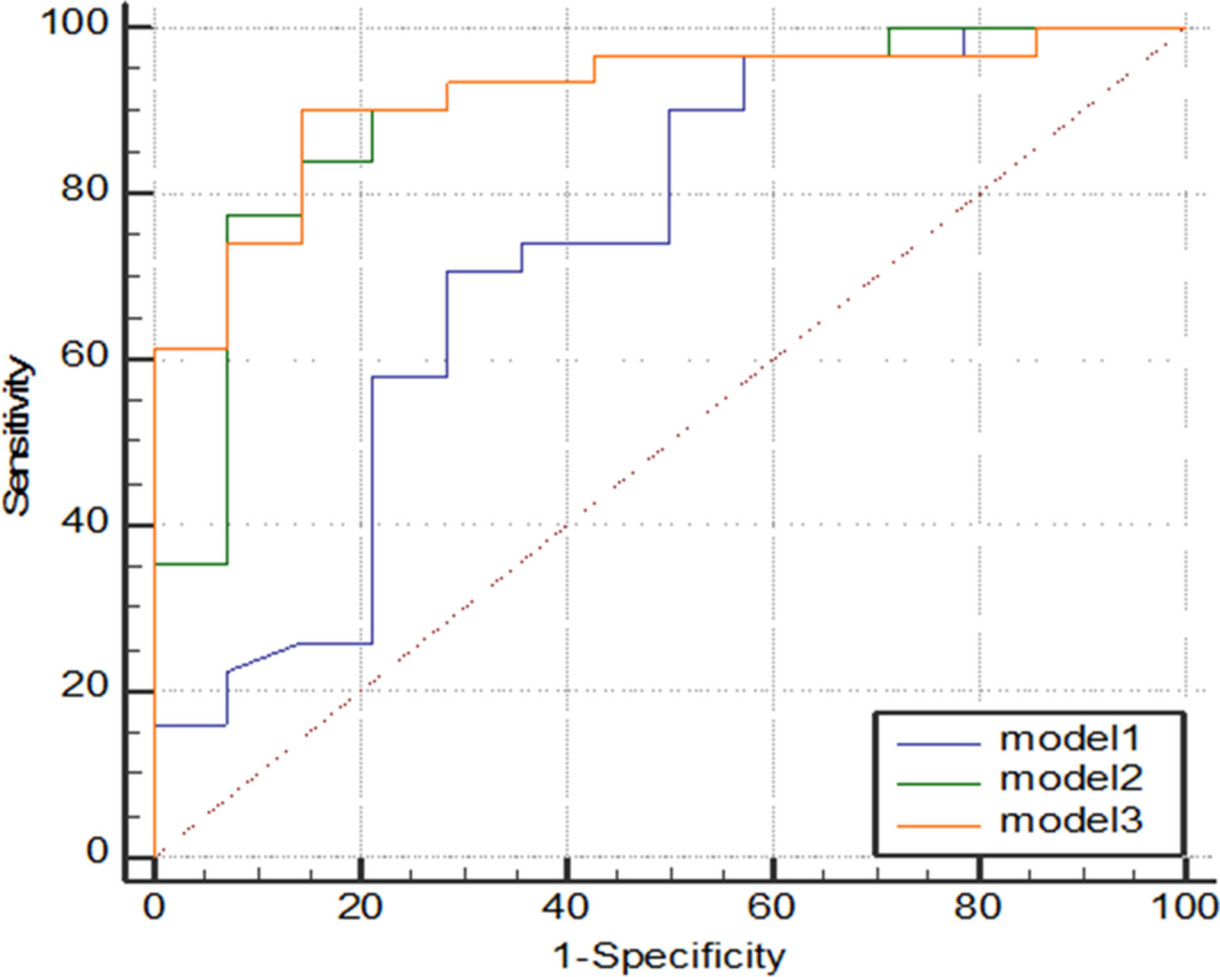
Graph shows comparison of ROC curves among three models Model 3 resulted in significantly highest AUC, indicating adding quantitative DCE-MRI parameters to model 1 and model 2 significantly improved diagnostic performance.

The multivariate model of conventional DCE-MRI features (model 1) resulted in an area under the curve (AUC) value of 0.63. The diagnostic model 2 that included the conventional DCE-MRI parameters plus ADC values performs obviously better (AUC of 0.89) than model 1 before ADC was added (*P* = 0.039). The diagnostic model 3 that included the conventional DCE-MRI parameters, ADC values, and quantitative DCE-MRI parameters performs obviously better than model 1 (*P* = 0.027) and clearly better than model 2 (*P* = 0.486). Model 3 also yielded a significantly highest AUC value of 0.92 (Figure 3).

### Pathological biomarkers of the two subtypes of breast cancer

**Figure 4:**
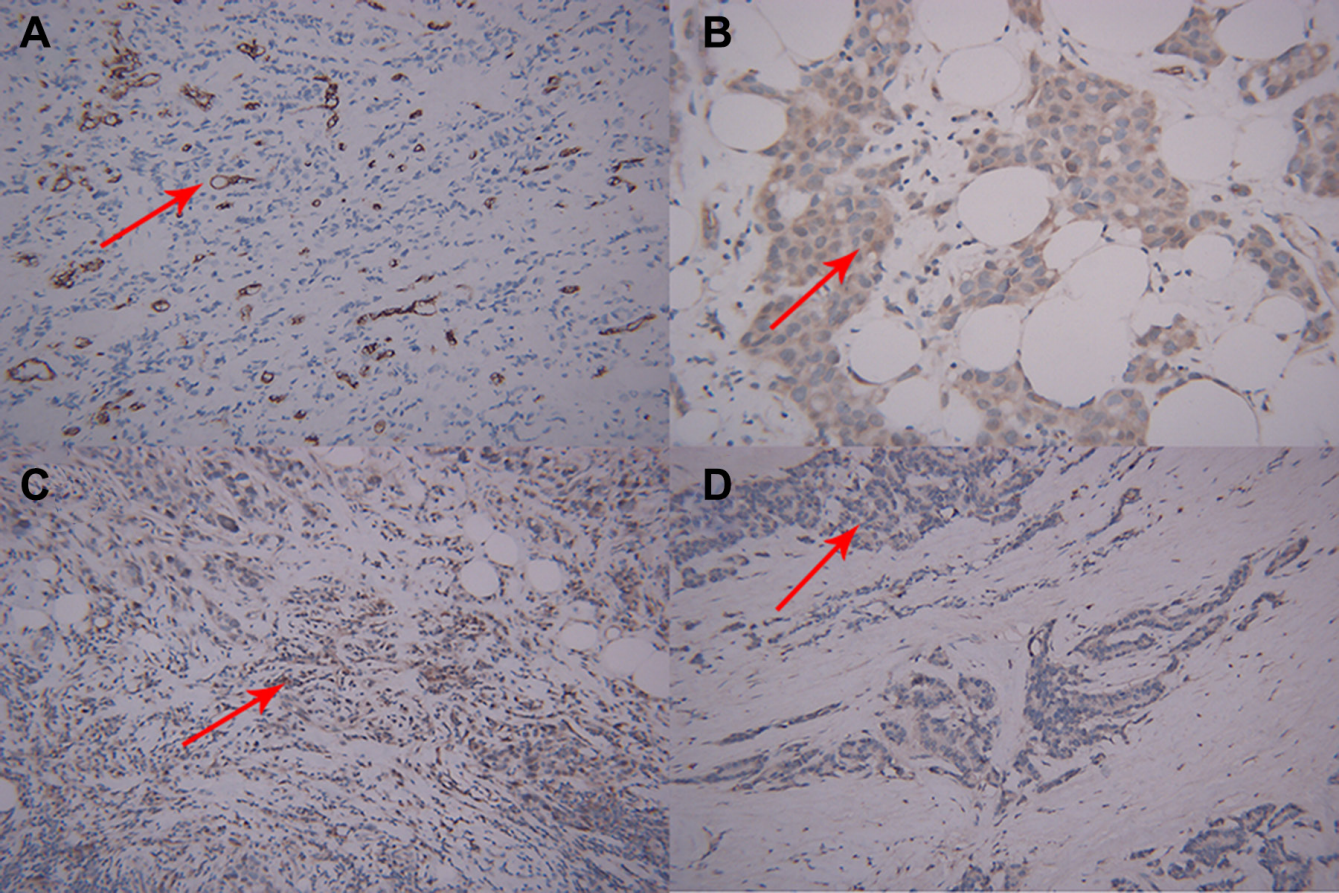
Microscopic manifestations of IHC in a patient with breast cancer same to Figure 1 Expression of biological factors: (**A**) high MVD distribution (CD34-immunostaining, original magnification ×200); (**B**) high VEGF expression (IHC, original magnification ×200); (**C**) high MMP-9 expression (IHC, original magnification ×200); (**D**) high MMP-2 expression (IHC, original magnification ×200).

**Figure 5:**
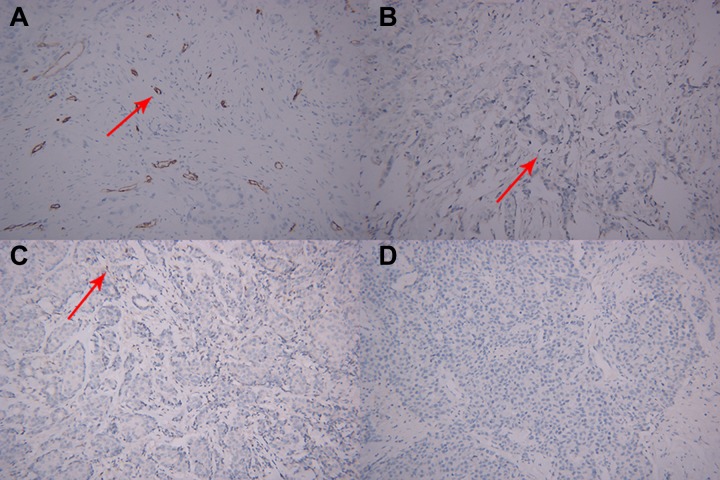
Microscopic manifestations of IHC in a patient with breast cancer same to Figure 2 (**A**) Low MVD distribution (CD34-immunostaining, original magnification ×200); (**B**) low VEGF expression (IHC, original magnification ×200); (**C**) low MMP-9 expression (IHC, original magnification ×200); (**D**) low MMP-2expression (IHC, original magnification ×200).

Table 4 shows that low-risk breast cancers exhibited significantly lower MMP-2 expression level (*P* = 0.000), lower MMP-9 expression level (*P* = 0.001), and lower MVD values (*P* = 0.008) compared with non-low-risk breast cancers. In contrast to non-low-risk breast cancer, low-risk breast cancer exhibited a lower VEGF-1 expression level. However, the differences between the two subtypes are not statistically significant (*P* = 0.102). Figure 4 shows a representative example of microscopic manifestations of IHC in a patient with non-low-risk breast cancer same to Figure 1. Figure 5 shows a representative example of microscopic manifestations of IHC in a patient with low-risk breast cancer same to Figure 2.

**Table 4:** Comparison of pathological biomarkers between two subtypes of breast cancer

Pathological biomarkers	Low-risk tumor (*n* = 40) Mean ± SD	Non-low-risk tumor(*n* = 64) Mean ± SD	*t*	*P*
MVD	71.85 ± 12.071	113.47 ± 9.401	2.746	0.008^*^
VEGF-1	3.05 ± 0.420	3.90 ± 0.308	1.668	0.102
MMP-2	0.65 ± 0.319	4.13 ± 0.328	7.276	0.000^*^
MMP-9	2.45 ± 0.456	4.37 ± 0.294	3.708	0.001^*^

Note—MVD means microvessel density; VEGF means vascular endothelial growth factor; MMP means matrix metalloproteinase.

SD means standard deviation.

Significant differences on the 0.05 level are marked by ^*^.

## DISCUSSION

Among women, one of the leading reasons of death is breast cancer [9]. In the United States, much more than 234,000 new breast cancer are diagnosed every year [10]. Bleyer et al. [11] reported that about 30% of the breast tumors would be overdiagnosed and treated in the United States. Breast carcinoma with its histological appearance, biomarker expression, and prognosis is a heterogeneous disease [12]. Han et al. reported that omission of RT becomes a standard operation for luminal A breast cancer [13, 14]. In clinical practice, it is of great importance to distinguish the low-risk breast cancers from aggressive cancers. Therefore, before operation, it is practical and useful to make risk stratification, based on the MR imaging in the clinical setting. MRI examination is known for its inherent high sensitivity. Nonspecific imaging features as well as the dependence on the expertise of the reader is the limitation of conventional MRI [15]. The parameters of semiquantitative DCE-MRI are limited by different factors, such as measurement settings dependence, MRI protocols, and an unclear interpretation of hemodynamic parameters [16]. DWI has a higher specificity but lower sensitivity [17]. A few reports evaluated the utility of the combined conventional DCE-MRI and DWI and its clinical application [17–19]. However, the clinical value of the combined DWI and DCE-MRI for breast lesions remains unclear and is controversial. The potential advantages of quantitative DCE-MRI analysis include a more meaningful representation of tissue physiology and a theoretic reproducibility that is less influenced by individual hemodynamic fluctuations, MRI protocols, and scanner settings. This approach has been successfully applied for perfusion and permeability measurements in brain tissue [20], kidney [21] and osteoporosis [22]. Thus, this study explored quantitative DCE-MRI alone or its combination with conventional DCE-MRI and DWI in the identification of low-risk breast cancer before operation.

Our results revealed significant differences in quantitative DCE-MRI parameters and ADC values exist between the two subtypes. Low-risk breast cancers show lower *K*^trans^, *K*_ep_, but higher ADC value than non-low-risk ones. Higher *K*^trans^, *K*_ep_, as well as lower Ve can be observed in triple-negative breast cancer [8, 23, 24]. Published reports showed that DCE-MRI improved specificity over the conventional breast MRI, with high sensitivity [8, 25, 26]. An negative correlation between ADC values and tumor cellularity has been described, indicating the relationship between proliferation rate and tumor aggressiveness [27, 28]. Low-risk breast cancers are considered to be lower aggressiveness than non-low-risk breast cancer. Their properties of biomarker Ki67 < 15% also show that they have lower cellular proliferation than non-low-risk breast cancers do [4, 6]. So, low-risk breast cancers displayed higher ADC values than the non-low-risk ones.

Meanwhile, there were no significant differences in the clinic-pathological data and conventional DCE-MRI findings between the two subtypes of breast cancers, indicating their inability to identify low-risk tumors. In our results, ROC curve analysis revealed that the diagnostic model including DCE-MRI and ADC values performed significantly better than the conventional DCE-MRI alone. Furthermore, the diagnostic model including conventional MRI parameters, ADC values, and quantitative DCE-MRI parameters yielded the highest AUC. This finding indicated that the *K*^trans^, *K*_ep_, and ADC values were important parameters in improving the diagnostic performance of low-risk breast cancers.

In the present research, some pathological biomarkers of the two subtypes of breast cancer, such as microvessel density (MVD), matrix metalloproteinase (MMP)-2, MMP-9 and vascular endothelial growth factor (VEGF-1)expression levels, were evaluated to explore the pathological basis of quantitative DCE-MRI and ADC value. The perfusion parameter values of tumors were decided not only by the MVD of tumors but also by other factors including the microvascular structure, the size of the extracellular space, and the vascular permeability of tumors. In the evaluation of tumor angiogenesis, MVD value has been considered as the standard. VEGF is an important proangiogenic molecule because it increases endothelial cell growth, blood vessel permeability, cell migration, and cell differentiation [29]. Meanwhile, the MMP family are actively connected with biological changes, such as embryogenesis, and bone regeneration, from which MMP-9 and MMP-2 particularly works in the breast tumor [30, 31]. The results of this study show that low-risk tumors had less MVD and lower VEGF, MMP-2, and MMP-9 expression levels compared with non-low-risk breast cancers. The study of Sabatier et al. showed that triple-negative breast carcinomas are characterized by higher MVD compared with the non-triple-negative breast carcinomas [32]. Liu et al. reported that VEGF immunoreactivities were correlated with the tumor stage, histological grade, and nodal involvement. VEGF expression was always commonly detected in HER2, luminal B, as well as luminal A tumors versus basal-like tumors [33]. Mira et al. reported that, compared with indolent tumor, the activity and expression level of MMP-9 are higher in invasive tumor [34]. The perfusion parameters and ADC value were closely associated with tumor angiogenesis. The fact that low-risk cancers showed lower *K*^trans^ and *K*_e*p*_ values was consistent with their less MVD and lower VEGF-1, MMP-2, and MMP-9 expression levels. Our study showed that low-risk breast tumors had elevated ADC value than that of non-low-risk ones. Low-risk breast cancers demonstrated lower MVD, VEGF, MMP-2 and MMP-9 than non-low-risk breast cancers. A negative correlation between the tumor ADC values and these pathological biomarkers was found. Thus low-risk breast cancers were assumed to be less hypervascular. On the other hand, Tumor proliferation can be quantified with Ki67 immunohistochemistry [35]. Low-risk breast cancers display lower expression of Ki67 than non-low-risk breast cancers based on its definition, indicating they hold a characteristic of sparse cellular density. Recent reports have shown that the ADC values are strongly correlated with the tissue cellularity inversely [36]. Those factors perhaps are the pathological basis of ADC value difference between two subtypes of breast cancer.

This research has some insufficient. Firstly, the number of samples is not greatly enormous. A larger sample size is necessary to provide further evidence to draw a valid conclusion. Second, only partial consistency was observed between the ROI selection of DCE-MRI and the pathological location. Given the heterogeneity of breast cancer, the process may induce mismatch in the relationship between imaging findings and pathological results, although extraction of the parenchyma was attempted during ROI outline and sample treatment. Third, the two-chamber Tofts model may not reflect the true condition of breast cancer tissue in vascular space and permeability. With the improvement of the software and hardware of MRI, the quantitative DCE-MRI parameters would be more precise and accurate.

In conclusion, lower *K*^trans^, *K*_ep_ as well as higher ADC were observed in low-risk breast tumors. The combination of conventional DCE-MRI and its quantitative parameters plus DWI facilitates the diagnosed of low-risk cancers. Pathologically less MVD and lower expression levels of VEGF-1, MMP-2, and MMP-9 are perhaps relative to the imaging findings. These results may be used as imaging biomarkers to make a precise subtype diagnosis of breast cancer before operation to guide the treatment plan.

## MATERIALS AND METHODS

### Patient population

The MR images of 104 patients (age range, 22 years to 84 years; 57.46 ± 11.53 years old) with pathologically proven breast cancer between October 2012 and April 2016 were prospectively analyzed. All of included patients were Chinese woman. Any case with remote metastases was excluded in our study. Approval from the institutional review board was obtained, and informed consent was acquired before the patients underwent MRI examination.

### Breast MRI examination

All of the patients underwent breast MR examination in a 3.0 Tesla MR device (Verio, Siemens, Erlangen, Germany) with a gradient strength of 45 mT/ms and a gradient slew rate of 200 mT/ms (16 channels) in the department. A conventional breast MRI protocol was performed using a standard breast coil in the prone position. First, T1-weighted imaging (TR, 4.33 ms; TE, 1.48 ms; section thickness, 3 mm; matrix 384 × 384; NEX 2; and field of view (FOV) adjusted according to the breast volume) and T2-weighted/FS imaging (TR, 4,300 ms; TE, 61 ms; section thickness, 3 mm; FOV, 340 × 100 mm; matrix 320 × 98; NEX 2) of bilateral breasts in the axial plane were acquired using a fast spin echo sequence. Spectral attenuation with inversion recovery was used for fat suppression. Second, axial DW images were obtained using a spin echo single-shot echo-planar imaging sequence (TR, 7,500 ms; TE, 81 ms; section thickness, 3 mm; FOV, 340 × 38 mm; matrix 192 × 85; NEX 3; *b* values were selected as 50 s/mm^2^, 400 s/mm^2^, and 800 s/mm^2^). Isotropic DWIs (trace) were reconstructed for each *b* factor. Finally, for DCE-MRI, a T1-weighted fat-suppressed 3D fast low angle shot sequence FLASH was performed following contrast injection. The parameters included TR/TE, 4.33/1.48 ms; flip angle, 10°; section thickness, 1.1 mm; and number of slices, 144. A total of 35 acquisitions were obtained per patient. The temporal resolution of DCE-MRI was 10 s to 11 s for each acquisition. At last, delayed contrast enhanced MR imaging was performed with three dimensional volumetric interpolated breath-hold examination sequence (TR/TE, 7.1/2.05; section thickness, 1.5 mm; one signal average).The total scan duration after bolus injection was approximately 420 s. Bolus injection (4 mL/s) of the contrast agent gadopentetate dimeglumine (Magnevist, Bayer Schering, Berlin, Germany; concentration, 0.5 mol/L) was performed (dose, 0.1 mmol/kg), followed by 50 mL saline flush through the antecubital vein at the beginning of the third acquisition.

### Breast cancer MRI analysis

Breast MRI analysis was retrospectively reviewed by two experienced radiologists, who were blinded to the clinical and pathological information.

Conventional MRI analysis: Morphological assessment was performed using the Breast Imaging Reporting and Data System Lexicon [37]. The lesion shape (round, oval, or irregular), margin (smooth, irregular, or speculated), and enhancement pattern (homogeneous, heterogeneous, or rim) of the breast lesions were recorded.

DWI analysis: For the quantitative analysis of the data acquired from DWI, ADC maps were constructed automatically by the software installed in the workstation (Siemens Healthcare). Breast lesions on DW images were identified, and at least three regions of interest (ROIs) were manually positioned over the lesion on the ADC maps to avoid cystic, necrotic, or hemorrhagic components and the average values were obtained to be the final ADC value.

Quantitative DCE-MRI analysis: An imaging workstation (Syngo multimodality workplace software version B17_43.1_1.0; Siemens Healthcare) was used for offline post-processing of DCE-MRI data on the basis of the two-compartment Tofts model. The ROIs were placed in the obviously enhanced areas to avoid areas of non-enhancement within the lesion which should be consistent with the ROIs in DWI analysis in location and size. The tumor enhancement patterns of the time–signal intensity curve (TIC) and the DCE-MRI perfusion parameters were analyzed by the Tissue4D^®^ software. The TIC was generated from the discrete time–signal intensity points within the ROI, which were classified into three types: (1) the persistent pattern (type I), with a continuous increase in the signal intensity over time; (2) the plateau pattern (type II), in which the signal intensity does not change after its initial increase; and (3) the washout pattern (type III), with an initial increase and reaching the highest point, followed by a decrease in the signal intensity. Three quantitative DCE-MRI parameters, namely, volume transfer constant (*K*^trans^; in this case, between blood plasma and extravascular extracellular space), volume of extravascular extracellular space per unit volume of tissue (Ve), and rate constant between extravascular extracellular space and blood plasma (*K*_ep_), were calculated according to the method previously described in [22].

### Immunohistochemical staining analysis

In fact all breast mass was labeled by making a releasable suture at the upper outer corner of the mass during operation. Then the samplings performed by pathologists could try to be consistent with the location of image findings.

Microvessel density (MVD) was evaluated by counting anti-CD34 positive microvessels using a light microscope .The manual counts were carried out on the images according to Weidner et al criteria [38]. After scanning the entire tumor section with a light microscope under a low magnification (×40), the area with the highest number of microvessels was identified as a “hot spot” and microvessels were counted using a higher magnification (×200) on the area. Any brown-stained single endothelial cell or endothelial cell clusters separated from surrounding tumor cells and connective tissue elements were regarded as microvessel. Vascular endothelial growth factor-1 (VEGF-1), matrix metalloproteinase (MMP)-2 and MMP-9 expression levels were determined through immunohistochemical staining.

Membrane or cytoplasmic brown products were regarded as a positive value, 10 high-power fields were observed randomly, and 100 cells were counted per field to evaluate the ratio of stained cells and staining intensity semiquantitatively. Then, comprehensive assessment was conducted, as follows:

(1) The ratio of stained cells was scored as follows: 0 = no stained cells in any microscopic field, 1 = 1% to 10% of tumor cells stained positively, 2 = 11% to 50% of tumor cells stained positively, and 3 = 51% to 100% of tumor cells stained positively.
(2) The intensity of the stain was scored on the following scale: 0 = no staining, 1 = mild staining, 2 = moderate staining, and 3 = intense staining.
(3) Overall scores were assessed as follows: overall scores = scores of stained cell ratio plus scores of staining intensity.

The median VEGF-1, MMP-2, and MMP-9 staining scores were selected as cutoff values to categorize the tumors into lowly (0 to 3) and highly (4 to 6) expressed tumors.

### Statistical analysis

SPSS statistical software, version 20.0 (IBM SPSS, Armonk, NJ, USA) was used to perform statistical analysis. Fisher’s exact test and chi-square test were used to compare the MRI morphologic findings and pathological biomarkers (VEGF-1, MMP-2, and MMP-9) between two subtypes of breast cancer. Student’s *t* test was used to compare the quantitative data, such as perfusion parameters, ADC values, and MVD, between two subtypes breast cancer.

Logistic regression analysis was performed using the backward elimination method for all variables, resulting in three multivariate models. Model 1 is the conventional MRI diagnostic model including lesion size, shape, margin, and enhancement pattern. Model 2 included the conventional MRI parameters and ADC values. Model 3 included the conventional MRI parameters, ADC values and quantitative DCE-MRI parameters. Receiver operating characteristic (ROC) curve analysis was performed to assess the diagnostic performance of the three models in making diagnosis of low-risk and non-low-risk breast cancers before operation. Two-tailed statistical tests were consistently used, and *P* values less than 0.05 were considered statistically significant.

## Abbreviations

DCE-MRI: Dynamic contrast-enhancement magnetic
ADC: apparent diffusion coefficient
MVD: microvessel density
VEGF: vascular endothelial growth factor
MMP: matrix metalloproteinase
ROI: regions of interest
